# Some Novel Results Involving Prototypical Computation of Zagreb Polynomials and Indices for *SiO*_4_ Embedded in a Chain of Silicates

**DOI:** 10.3390/molecules28010201

**Published:** 2022-12-26

**Authors:** El Sayed M. Tag El Din, Faisal Sultan, Muhammad Usman Ghani, Jia-Bao Liu, Sanaullah Dehraj, Murat Cancan, Fahad M. Alharbi, Abdullah Alhushaybari

**Affiliations:** 1 Faculty of Engineering and Technology, Future University in Egypt, New Cairo 11835, Egypt; 2 Institute of Mathematics, Khawaja Fareed University of Engineering & Information Technology, Abu Dhabi Road, Rahim Yar Khan 64200, Pakistan; 3 School of Mathematics and Physics, Anhui Jianzhu University, Hefei 230601, China; 4 Department of Mathematics and Statistics, Quaid-e-Awam University of Engineering, Science and Technology, Sakrand Road, Nawabshah 67480, Pakistan; 5 Faculty of Education, VAN Yuzuncu Yil University, Van 65090, Turkey; 6 Department of Mathematics, Al-Qunfudah University College, Umm Al-Qura University, Mecca 21421, Saudi Arabia; 7 Department of Mathematics, College of Science, Taif University, P.O. Box 11099, Taif 21944, Saudi Arabia

**Keywords:** *SiO*_4_ embedded in a chain of silicates, zagreb polynomials, zagreb indices

## Abstract

A topological index as a graph parameter was obtained mathematically from the graph’s topological structure. These indices are useful for measuring the various chemical characteristics of chemical compounds in the chemical graph theory. The number of atoms that surround an atom in the molecular structure of a chemical compound determines its valency. A significant number of valency-based molecular invariants have been proposed, which connect various physicochemical aspects of chemical compounds, such as vapour pressure, stability, elastic energy, and numerous others. Molecules are linked with numerical values in a molecular network, and topological indices are a term for these values. In theoretical chemistry, topological indices are frequently used to simulate the physicochemical characteristics of chemical molecules. Zagreb indices are commonly employed by mathematicians to determine the strain energy, melting point, boiling temperature, distortion, and stability of a chemical compound. The purpose of this study is to look at valency-based molecular invariants for $SiO_{4}$ embedded in a silicate chain under various conditions. To obtain the outcomes, the approach of atom–bond partitioning according to atom valences was applied by using the application of spectral graph theory, and we obtained different tables of atom—bond partitions of $SiO_{4}$. We obtained exact values of valency-based molecular invariants, notably the first Zagreb, the second Zagreb, the hyper-Zagreb, the modified Zagreb, the enhanced Zagreb, and the redefined Zagreb (first, second, and third). We also provide a graphical depiction of the results that explains the reliance of topological indices on the specified polynomial structure parameters.

## 1. Introduction

A molecular structure is defined as a simple and linked network *G*, where |G| is the set of atoms (nodes) and $V_{G}$ is the set of atom–bonds (links between atoms) [1]. If two atoms ${\dot{a}}_{1}$ and ${\dot{a}}_{2}$ form an atom–bond in *G*, we write ${\dot{a}}_{1}∼{\dot{a}}_{2}$; similarly, if two atoms do not form an atom–bond in *G*, we write ${\dot{a}}_{1}≁{\dot{a}}_{2}$. The topological index of a chemical composition is a numerical value or a continuation of a given structure under discussion, which indicates chemical, physical, and biological properties of a chemical molecule, see for details [2,3,4]. Topological indices and polynomials capture molecular structural symmetries and provide mathematical vocabulary for predicting features, such as boiling temperatures, viscosity, radius of gyrations, and so on [5,6].

Mathematical chemistry describes how to use polynomials and functions to offer instructions concealed in the symmetry of molecular graphs, and the graph theory has many applications in modern chemistry, particularly organic chemistry. In chemical graph theory, the atoms and bonds of a molecular structure are represented by vertices and edges, respectively [7]. Many applications of topological indices are employed in theoretical chemistry, particularly in research pertaining to quantitative structure–property relationships (QSPRs) and quantitative structure–activity relationships (QSARs) [8,9,10]. Many famous researchers have studied topological indices to obtain information about different families of graphs [11,12]. In (QSPR) and (QSAR), topological indices are utilized directly as simple numerical descriptors in comparison with physical, biological, and chemical characteristics of molecules, which are benefits. Many researchers have worked on various chemical compounds and computed topological descriptors of various molecular graphs during the last few decades [13,14].

The molecular graph is a simple connected graph in a chemical graph theory that contains chemical atoms and bonds, which are often referred to as vertices and edges, respectively, and there must be a linkage between the vertex set $V_{G}$ and edge set $E_{G}$. The valency of each atom of *G* is actually the total number of atoms connected to *v* of *G* and is denoted by $d_{v}$, [15].

In 1972, Gutman and Trinajstic initiated the idea of computing the branching of the carbon–atom skeleton, which was, later on, known as the first Zagreb index [16]. In 2004, Gutman and Das, adulated characteristics of the first and second Zagreb polynomials for chemical graphs of a chemical compound, which we studied in the research articles [17]. The first Zagreb polynomial corresponding to the first Zagreb index is defined as

(1) $$M_{1}\left(G,y\right)=\sum_{uv\in E_{G}}y^{d_{u}+d_{v}}\;\;\;\&\;\;\;M_{1}\left(G\right)=\sum_{u,v\in E_{G}}d_{u}+d_{v}$$

The second Zagreb polynomial, which corresponds to the second Zagreb index [17], is written as

(2) $$M_{2}\left(G,y\right)=\sum_{u,v\in E_{G}}y^{d_{u}d_{v}}\;\;\;\&\;\;\;M_{2}\left(G\right)=\sum_{u,v\in E_{G}}d_{u}d_{v}$$

In 2013, Shirdel et al. initiated the concept of the hyper-Zagreb index [18]. The hyper-Zagreb polynomial and index are defined as follows:

(3) $$HM\left(G,y\right)=\sum_{u,v\in E_{G}}y^{{\left(d_{u}+d_{v}\right)}^{2}}\;\;\;\&\;\;\;HM\left(G\right)=\sum_{u,v\in E_{G}}{\left(d_{u}+d_{v}\right)}^{2}$$

The modified Zagreb polynomial and index [19] are defined as

(4) $$MD\left(G,y\right)=\sum_{u,v\in E_{G}}y^{\frac{1}{d_{u}d_{v}}}\;\;\;\&\;\;\;MD\left(G\right)=\sum_{u,v\in E_{G}}\frac{1}{d_{u}d_{v}}$$

In 2010, Furtula et al. introduced the augmented Zagreb index [20]. The augmented Zagreb polynomial and index are defined as

(5) $$AZI\left(G,y\right)=\sum_{u,v\in E_{G}}y^{{\left[\frac{\left(d_{u}d_{v}\right)}{\left(d_{u}+d_{v}-2\right)}\right]}^{3}}\;\;\;\&\;\;\;AZI\left(G\right)=\sum_{u,v\in E_{G}}{\left[\frac{\left(d_{u}d_{v}\right)}{\left(d_{u}+d_{v}-2\right)}\right]}^{3}$$

In 2013, Ranjini, Lokesha, and Usha presented [21] a redesigned version of the Zagreb indices ReZG1, ReZG2, and ReZG3. The indices and redefined form of the Zagreb polynomial are as follows:

(6) $$ReZG_{1}\left(G,y\right)=\sum_{u,v\in E_{G}}y^{\frac{d_{u}+d_{v}}{d_{u}d_{v}}}\;\;\;\&\;\;\;ReZG_{1}=\sum_{u,v\in E(G)}\frac{d_{u}+d_{v}}{d_{u}d_{v}}$$

(7) $$ReZG_{2}\left(G,y\right)=\sum_{u,v\in E_{G}}y^{\frac{d_{u}d_{v}}{d_{u}+d_{v}}}\;\;\;\&\;\;\;ReZG_{2}=\sum_{u,v\in E_{G}}\frac{d_{u}d_{v}}{d_{u}+d_{v}}$$

(8) $$ReZG_{3}\left(G,y\right)=\sum_{u,v\in E_{G}}y^{\left(d_{u}d_{v}\right)\left(d_{u}+d_{v}\right)}\;\;\;\&\;\;\;ReZG_{3}=\sum_{u,v\in E_{G}}\left(d_{u}d_{v}\right)\left(d_{u}+d_{v}\right)$$

In this article, the above-defined eight Zagreb polynomials and Zagreb indices were constructed by the atom–bond set of silicates, partitioned according to the valencies of the $S_{i}$ and $O_{2}$ atoms, [22]. We also investigate silicon tetrahedron $S_{i}O_{4}$ in a compound structure and derived the precise formulas of certain essential valency-based Zagreb indices using the approach of the atom–bond partitioning of the molecular structure of silicates; for details, see [23,24].

## 2. Chain of Silicates

**Figure 1:**
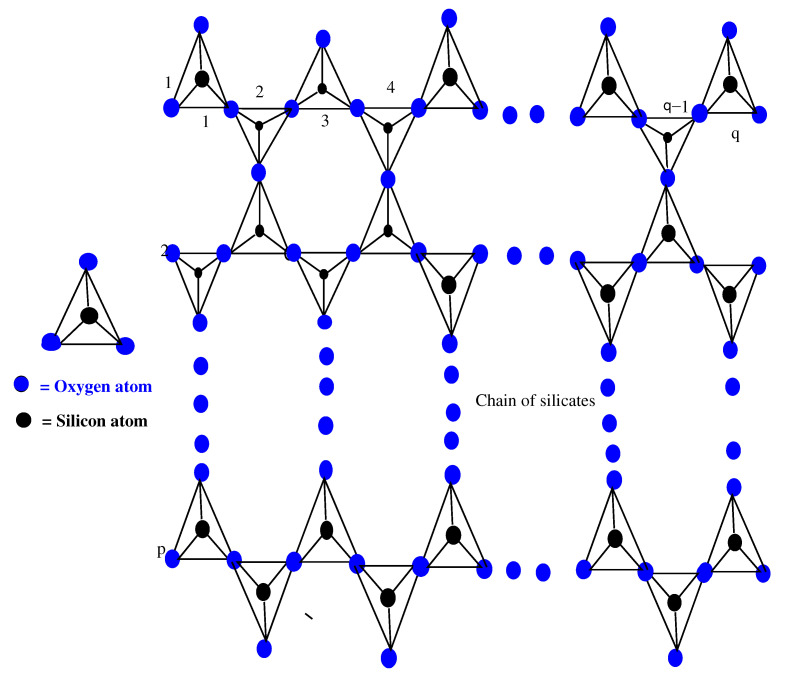
Chain of $SiO_{4}$.

The basic unit of silicates is a $SiO_{4}$ tetrahedron, which is obtained by metal carbonates with sand or fusing metal oxides [25]. Almost all of the silicates contain $SiO_{4}$ tetrahedron. From a chemical point of view, for a tetrahedron $SiO_{4}$, we consider a pyramid with a triangular base (single tetrahedron $SiO_{4}$), as shown in Figure 1, containing oxygen atoms $O_{2}$ at the four corners of the tetrahedron, and the silicon atom $S_{i}$ is bonded with equally spaced atoms of $O_{2}$. From the resulting $SiO_{4}$, a silicate tetrahedron joins with other $SiO_{4}$ horizontally, and a single chain of silicates is obtained. Similarly, when two molecules of $SiO_{4}$ join corner-to-corner, then each $SiO_{4}$ shares its $O_{2}$ atom with the other $SiO_{4}$ molecule, as seen in Figure 1. After completing this process of sharing, these two molecules of $SiO_{4}$ can be joined with two other molecules. Now, we obtain a chain of silicates $SC_{q}^{p}$, where *p* and *q* are the silicate chain numbers formed and the total number of $SiO_{4}$ in one silicate chain, respectively. Here, in the chain of silicates $SC_{q}^{p}$, pq is the number of tetrahedron $SiO_{4}$ used, see Figure 1.

Here, in the chain of silicates $SC_{q}^{p}$, there are three types of atom–bonds on the basis of valency of every atom of $SC_{p}^{p}$. Therefore, there are two types of atoms, $v_{i}$ and $v_{j}$, such that $d_{v_{i}}=3$ and $d_{v_{j}}=6$, where $d_{v_{i}}$ and $d_{v_{j}}$ mean the valencies of atoms ∀$v_{i},v_{j}\in SC_{p}^{p}$. According to valencies 3 and 6 of the atoms, there are three types of atom–bonds, which are (3∼3), (3∼6), and (6∼6) in $SC_{q}^{p}$. On the basis of valency, Table 1 provides the partition of the set of atom–bonds.

## 3. Zagreb Polynomials and Indices for p,q≥ 2, p=q

**Theorem** **1.** *For p>1, the first Zagreb polynomial of $SC_{p}^{p}$ is $\left(3p+2\right)y^{6}+\left(3p^{2}+3p-4\right)y^{9}+\left(3p^{2}-6p+2\right)y^{12}$.*

**Proof.**  Using the atom–bond partition from Table 1, in the formula of the first Zagreb polynomial (1), we have

$$\begin{array}{ccc} M_{1}\left(SC_{p}^{p},y\right) & = & \sum_{E_{3∼3}}y^{3+3}+\sum_{E_{3∼6}}y^{3+6}+\sum_{E_{6∼6}}y^{6+6} \end{array}$$

This gives

$$\begin{array}{ccc} M_{1}\left(SC_{p}^{p},y\right) & = & \left(3p+2\right)y^{6}+\left(3p^{2}+3p-4\right)y^{9}+\left(3p^{2}-6p+2\right)y^{12}. \end{array}$$

□

By taking the first derivative of the polynomial in Theorem 1 at y=1, we obtain the first Zagreb index of the silicate network $SC_{p}^{p}$ as follows: For p>1, the first Zagreb index of $SC_{p}^{p}$ is $63p^{2}-27p$.

**Theorem** **2.** *For p>1, the second Zagreb polynomial of $SC_{p}^{p}$ is $\left(3p+2\right)y^{9}+\left(3p^{2}+3p-4\right)y^{18}+\left(3p^{2}-6p+2\right)y^{36}$.*

**Proof.**  Using the atom–bond partition from Table 1, in the formula of the second Zagreb polynomial (2), we have

$$\begin{array}{ccc} M_{2}\left(SC_{p}^{p},y\right) & = & \sum_{E_{3∼3}}y^{3\times 3}+\sum_{E_{3∼6}}y^{3\times 6}+\sum_{E_{6∼6}}y^{6\times 6} \end{array}$$

This gives

$$\begin{array}{ccc} M_{2}\left(SC_{p}^{p},y\right) & = & \left(3p+2\right)y^{9}+\left(3p^{2}+3p-4\right)y^{18}+\left(3p^{2}-6p+2\right)y^{36}. \end{array}$$

□

By taking the first derivative of the polynomial in Theorem 2 at y=1, we obtain the second Zagreb index of the chain of silicates $SC_{p}^{p}$ as follows: For p>1, the second Zagreb index of $SC_{p}^{p}$ is $162p^{2}-1135p+18$.

**Theorem** **3.** *For p>1, the hyper-Zagreb polynomial of $SC_{p}^{p}$ is $\left(3p+2\right)y^{36}+\left(3p^{2}+3p-4\right)y^{81}+\left(3p^{2}-6p+2\right)y^{144}$.*

**Proof.**  Using the atom–bond partition from Table 1, in the formula of the hyper-Zagreb polynomial (3), we have

$$\begin{array}{ccc} HM(SC_{p}^{p},y) & = & \sum_{E_{3∼3}}y^{{\left(3+3\right)}^{2}}+\sum_{E_{3∼6}}y^{{\left(3+6\right)}^{2}}+\sum_{E_{6∼6}}y^{{\left(6+6\right)}^{2}} \end{array}$$

This gives

$$\begin{array}{ccc} HM(SC_{p}^{p},y) & = & \left(3p+2\right)y^{36}+\left(3p^{2}+3p-4\right)y^{81}+\left(3p^{2}-6p+2\right)y^{144}. \end{array}$$

□

By taking the first derivative of the polynomial in Theorem 3 at y=1, we obtain the hyper-Zagreb index of the chain of silicates $SC_{p}^{p}$ as follows: For p>1, the hyper-Zagreb index of $SC_{p}^{p}$ is $675p^{2}+513p+36$.

**Theorem** **4.** *For p>1, the modified Zagreb polynomial of $SC_{p}^{p}$ is $\left(3p+2\right)y^{\frac{1}{9}}+\left(3p^{2}+3p-4\right)y^{\frac{1}{18}}+\left(3p^{2}-6p+2\right)y^{\frac{1}{36}}$.*

**Proof.**  Using the atom–bond partition from Table 1, in the formula of the modified Zagreb polynomial (4), we have

$$\begin{array}{ccc} MD(SC_{p}^{p},y) & = & \sum_{E_{3∼3}}y^{\frac{1}{3\times 3}}+\sum_{E_{3∼6}}y^{\frac{1}{3\times 6}}+\sum_{E_{6∼6}}y^{\frac{1}{6\times 6}} \end{array}$$

This gives

$$\begin{array}{ccc} MD(SC_{p}^{p},y) & = & \left(3p+2\right)y^{\frac{1}{9}}+\left(3p^{2}+3p-4\right)y^{\frac{1}{18}}+\left(3p^{2}-6p+2\right)y^{\frac{1}{36}}. \end{array}$$

□

By taking the first derivative of the polynomial in Theorem 4 at y=1, we obtain the modified Zagreb index of the chain of silicates $SC_{p}^{p}$ as follows: For p>1, the modified Zagreb index of $SC_{p}^{p}$ is $\frac{1}{4}p^{2}+\frac{1}{3}p+\frac{1}{18}$.

**Theorem** **5.** *For p>1, the augmented Zagreb polynomial of $SC_{p}^{p}$ is $\left(3p+2\right)y^{\frac{729}{64}}+\left(3p^{2}+3p-4\right)y^{\frac{5832}{343}}+\left(3p^{2}-6p+2\right)y^{\frac{5832}{125}}$.*

**Proof.**  Using the atom–bond partition from Table 1, in the formula of the augmented Zagreb polynomial (5), we have

$$\begin{array}{ccc} AZI(SC_{p}^{p},y) & = & \sum_{E_{3∼3}}y^{{\left(\frac{3\times 3}{3+3-2}\right)}^{3}}+\sum_{E_{3∼6}}y^{{\left(\frac{3\times 6}{3+6-2}\right)}^{3}}+\sum_{E_{6∼6}}y^{{\left(\frac{6\times 6}{6+6-2}\right)}^{3}} \end{array}$$

This gives

$$\begin{array}{ccc} AZI(SC_{p}^{p},y) & = & \left(3p+2\right)y^{\frac{729}{64}}+\left(3p^{2}+3p-4\right)y^{\frac{5832}{343}}+\left(3p^{2}-6p+2\right)y^{\frac{5832}{125}}. \end{array}$$

□

By taking the first derivative of the polynomial in Theorem 5 at y=1, we obtain the augmented Zagreb index of the chain of silicates $SC_{p}^{p}$ as follows: For p>1, the augmented Zagreb index of $SC_{p}^{p}$ is $\frac{8188128}{42875}p^{2}-\frac{53440879}{2744000}p+\frac{65967939}{1372000}$.

**Theorem** **6.** *For p>1, the first redefined Zagreb polynomial of $SC_{p}^{p}$ is $\left(3p+2\right)y^{\frac{2}{3}}+\left(3p^{2}+3p-4\right)y^{\frac{1}{2}}+\left(3p^{2}-6p+2\right)y^{\frac{1}{3}}$.*

**Proof.**  Using the atom–bond partition from Table 1, in the formula of the first redefined Zagreb polynomial (6), we have

$$\begin{array}{ccc} ReZG_{1}\left(SC_{p}^{p},y\right) & = & \sum_{E_{3∼3}}y^{\left(\frac{3+3}{3\times 3}\right)}+\sum_{E_{3∼6}}y^{\left(\frac{3+6}{3\times 6}\right)}+\sum_{E_{6∼6}}y^{\left(\frac{6+6}{6\times 6}\right)} \end{array}$$

This gives

$$\begin{array}{ccc} ReZG_{1}\left(SC_{p}^{p},y\right) & = & \left(3p+2\right)y^{\frac{2}{3}}+\left(3p^{2}+3p-4\right)y^{\frac{1}{2}}+\left(3p^{2}-6p+2\right)y^{\frac{1}{3}}. \end{array}$$

□

By taking the first derivative of the polynomial in Theorem 6 at y=1, we obtain the first redefined Zagreb index of the chain of silicates $SC_{p}^{p}$ as follows: For p>1, the first redefined Zagreb index of $SC_{p}^{p}$ is $\frac{5}{2}p^{2}+2p-\frac{1}{2}$.

**Theorem** **7.** *For p>1, the second redefined Zagreb polynomial of $SC_{p}^{p}$ is $\left(3p+2\right)y^{\frac{3}{2}}+\left(3p^{2}+3p-4\right)y^{2}+\left(3p^{2}-6p+2\right)y^{3}$.*

**Proof.**  Using the atom–bond partition from Table 1, in the formula of the second redefined Zagreb polynomial (7), we obtain

$$\begin{array}{ccc} ReZG_{2}\left(SC_{p}^{p},y\right) & = & \sum_{E_{3∼3}}y^{\left(\frac{3\times 3}{3+3}\right)}+\sum_{E_{3∼6}}y^{\left(\frac{3\times 6}{3+6}\right)}+\sum_{E_{6∼6}}y^{\left(\frac{6\times 6}{6+6}\right)} \end{array}$$

This gives

$$\begin{array}{ccc} ReZG_{2}\left(SC_{q}^{p},y\right) & = & \left(3p+2\right)y^{\frac{3}{2}}+\left(3p^{2}+3p-4\right)y^{2}+\left(3p^{2}-6p+2\right)y^{3}. \end{array}$$

□

By taking the first derivative of the polynomial in Theorem 7 at y=1, we obtain the second redefined Zagreb index of the chain of silicates $SC_{p}^{p}$ as follows: For p>1, the second redefined Zagreb index of $SC_{p}^{p}$ is $15p^{2}-\frac{34}{3}p+1$.

**Theorem** **8.** *For p>1, the third redefined Zagreb polynomial of $SC_{p}^{p}$ is $\left(3p+2\right)y^{54}+\left(3p^{2}+3p-4\right)y^{196}+\left(3p^{2}-6p+2\right)y^{432}$.*

**Proof.**  Using the atom–bond partition from Table 1, in the formula of the third redefined Zagreb polynomial (8), we obtain

$$\begin{array}{ccc} ReZG_{3}\left(SC_{p}^{p},y\right) & = & \sum_{E_{3∼3}}y^{\left(3\times 3)(3+3\right)}+\sum_{E_{3∼6}}y^{\left(3\times 6)(3+6\right)}+\sum_{E_{6∼6}}y^{\left(6\times 6)(6+6\right)} \end{array}$$

This gives

$$\begin{array}{ccc} ReZG_{3}\left(SC_{p}^{p},y\right) & = & \left(3p+2\right)y^{54}+\left(3p^{2}+3p-4\right)y^{196}+\left(3p^{2}-6p+2\right)y^{432}. \end{array}$$

□

By taking the first derivative of the polynomial in Theorem 8 at y=1, we obtain the third redefined Zagreb index of the chain of silicates $SC_{p}^{p}$ as follows: For p>1, the third redefined Zagreb index of $SC_{p}^{p}$ is $188p^{2}-1842p+188$.

### Comparison

**Figure 2:**
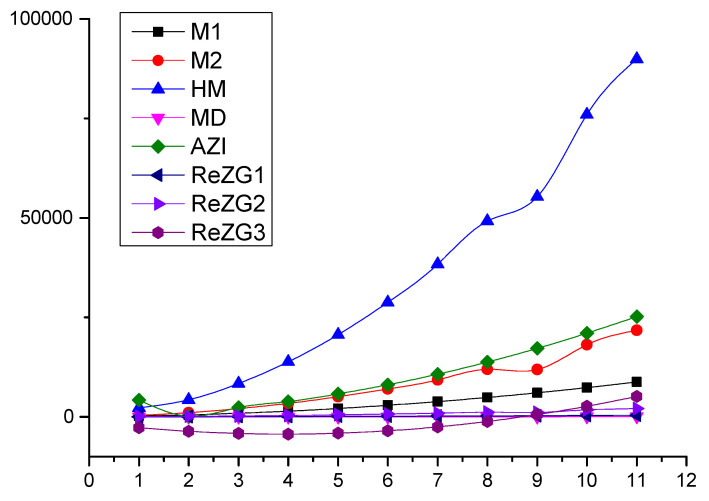
Graphical comparison of Zagreb indices for p,q≥ 2, p=q.

In this section, we present a numerical comparison of Zagreb indices in Table 2 and graphical comparison in Figure 2 of Zagreb polynomials for p,q>1 and p=q=2,3,4,…,12 for the chain of silicates $SC_{q}^{p}$.

## 4. Zagreb Polynomials and Indices for p<q and p Are Odd

Here, in the chain of silicates $SC_{q}^{p}$, we observed for p<q that p is odd and the atom–bond on the basis of the valency of every atom of $SC_{q}^{p}$ changed. So, on the basis of valency, Table 3 provides the partition of the set of atom–bonds.

**Theorem** **9.** *Let p be odd and p<q. Then the first Zagreb polynomial of $SC_{q}^{p}$ is $3\left(p+1\right)y^{6}+\left(3pq+p+2q-5\right)y^{9}+\left(3pq-4p-2q+2\right)y^{12}$.*

**Proof.**  Using the atom–bond partition from Table 3, in the formula of the first Zagreb polynomial (1), we obtain

$$\begin{array}{ccc} M_{1}\left(SC_{q}^{p},y\right) & = & \sum_{E_{3∼3}}y^{3+3}+\sum_{E_{3∼6}}y^{3+6}+\sum_{E_{6∼6}}y^{6+6} \end{array}$$

This gives

$$\begin{array}{ccc} M_{1}\left(SC_{q}^{p},y\right) & = & 3\left(p+1\right)y^{6}+\left(3pq+p+2q-5\right)y^{9}+\left(3pq-4p-2q+2\right)y^{12}. \end{array}$$

□

By taking the first derivative of the polynomial in Theorem 9 at y=1, we obtain the first Zagreb index of the silicate network $SC_{q}^{p}$ as follows: Let *p* be odd and p<q. Then the first Zagreb index of $SC_{q}^{p}$ is 63pq−216p−6q−3.

**Theorem** **10.** *Let p be odd and p<q. Then the second Zagreb polynomial of $SC_{q}^{p}$ is $3\left(p+1\right)y^{9}+\left(3pq+p+2q-5\right)y^{18}+\left(3pq-4p-2q+2\right)y^{36}$.*

**Proof.**  Using the atom–bond partition from Table 3, in the formula of the second Zagreb polynomial (2), we obtain

$$\begin{array}{ccc} M_{2}\left(SC_{q}^{p},y\right) & = & \sum_{E_{3∼3}}y^{3\times 3}+\sum_{E_{3∼6}}y^{3\times 6}+\sum_{E_{6∼6}}y^{6\times 6} \end{array}$$

This gives

$$\begin{array}{ccc} M_{2}\left(SC_{q}^{p},y\right) & = & 3\left(p+1\right)y^{9}+\left(3pq+p+2q-5\right)y^{18}+\left(3pq-4p-2q+2\right)y^{36}. \end{array}$$

□

By taking the first derivative of the polynomial in Theorem 10 at y=1, we obtain the second Zagreb index of the chain of silicates $SC_{q}^{p}$ as follows: Let *p* be odd and p<q. Then the second Zagreb index of $SC_{q}^{p}$ is 162pq−99p−36q+9.

**Theorem** **11.** *Let p be odd and p<q. Then the hyper-Zagreb polynomial of $SC_{q}^{p}$ is $3\left(p+1\right)y^{36}+\left(3pq+p+2q-5\right)y^{81}+\left(3pq-4p-2q+2\right)y^{144}$.*

**Proof.**  Using the atom–bond partition from Table 3, in the formula of the hyper-Zagreb polynomial (3), we obtain

$$\begin{array}{ccc} HM(SC_{q}^{p},y) & = & \sum_{E_{3∼3}}y^{36}+\sum_{E_{3∼6}}y^{81}+\sum_{E_{6∼6}}y^{144} \end{array}$$

This gives

$$\begin{array}{ccc} HM(SC_{q}^{p},y) & = & 3\left(p+1\right)y^{36}+\left(3pq+p+2q-5\right)y^{81}+\left(3pq-4p-2q+2\right)y^{144}. \end{array}$$

□

By taking the first derivative of the polynomial in Theorem 11 at y=1, we obtain the hyper-Zagreb index of the chain of silicates $SC_{q}^{p}$ as follows: Let *p* be odd and p<q. Then the hyper-Zagreb index of $SC_{q}^{p}$ is 675pq−387p−162q−9.

**Theorem** **12.** *Let p be odd and p<q. Then the modified Zagreb polynomial of $SC_{q}^{p}$ is $3\left(p+1\right)y^{\frac{1}{9}}+\left(3pq+p+2q-5\right)y^{\frac{1}{18}}+\left(3pq-4p-2q+2\right)y^{\frac{1}{36}}$.*

**Proof.**  Using the atom–bond partition from Table 3, in the formula of the modified Zagreb polynomial (4), we obtain

$$\begin{array}{ccc} MD(SC_{q}^{p},y) & = & \sum_{E_{3∼3}}y^{\frac{1}{3\times 3}}+\sum_{E_{3∼6}}y^{\frac{1}{3\times 6}}+\sum_{E_{6∼6}}y^{\frac{1}{6\times 6}} \end{array}$$

This gives

$$\begin{array}{ccc} MD(SC_{q}^{p},y) & = & 3\left(p+1\right)y^{\frac{1}{9}}+\left(3pq+p+2q-5\right)y^{\frac{1}{18}}+\left(3pq-4p-2q+2\right)y^{\frac{1}{36}}. \end{array}$$

□

By taking the first derivative of the polynomial in Theorem 12 at y=1, we obtain the modified Zagreb index of the chain of silicates $SC_{q}^{p}$ as follows: Let *p* be odd and p<q. Then the modified Zagreb index of $SC_{q}^{p}$ is $\frac{1}{4}pq+\frac{5}{8}p+\frac{1}{18}q+\frac{1}{9}$.

**Theorem** **13.** *Let p be odd and p<q. Then the augmented Zagreb polynomial of $SC_{q}^{p}$ is $3\left(p+1\right)y^{\frac{729}{64}}+\left(3pq+p+2q-5\right)y^{\frac{5832}{343}}+\left(3pq-4p-2q+2\right)y^{\frac{5832}{125}}$.*

**Proof.**  Using the atom–bond partition from Table 3, in the formula of the augmented Zagreb polynomial (5), we obtain

$$\begin{array}{ccc} AZI(SC_{q}^{p},y) & = & \sum_{E_{3∼3}}y^{{\left(\frac{3\times 3}{3+3-2}\right)}^{3}}+\sum_{E_{3∼6}}y^{{\left(\frac{3\times 6}{3+6-2}\right)}^{3}}+\sum_{E_{6∼6}}y^{{\left(\frac{6\times 6}{6+6-2}\right)}^{3}} \end{array}$$

This gives

$$\begin{array}{ccc} AZI(SC_{q}^{p},y) & = & 3\left(p+1\right)y^{\frac{729}{64}}+\left(3pq+p+2q-5\right)y^{\frac{5832}{343}}+\left(3pq-4p-2q+2\right)y^{\frac{5832}{125}}. \end{array}$$

□

By taking the first derivative of the polynomial in Theorem 13 at y=1, we obtain the augmented Zagreb index of the chain of silicates $SC_{q}^{p}$ as follows: Let *p* be odd and p<q. Then the augmented Zagreb index of $SC_{q}^{p}$ is $\frac{8188128}{42475}pq-\frac{1213056}{8192}p-\frac{2542752}{42875}q+\frac{116535753}{2744000}$.

**Theorem** **14.** *Let p be odd and p<q. Then the first redefined Zagreb polynomial of $SC_{q}^{p}$ is $3\left(p+1\right)y^{\frac{2}{3}}+\left(3pq+p+2q-5\right)y^{\frac{1}{2}}+\left(3pq-4p-2q+2\right)y^{\frac{1}{3}}$.*

**Proof.**  Using the atom–bond partition from Table 3, in the formula of the first redefined Zagreb polynomial (6), we obtain

$$\begin{array}{ccc} ReZG_{1}\left(SC_{q}^{p},y\right) & = & \sum_{E_{3∼3}}y^{\left(\frac{3+3}{3\times 3}\right)}+\sum_{E_{3∼6}}y^{\left(\frac{3+6}{3\times 6}\right)}+\sum_{E_{6∼6}}y^{\left(\frac{6+6}{6\times 6}\right)} \end{array}$$

This gives

$$\begin{array}{ccc} ReZG_{1}\left(SC_{q}^{p},y\right) & = & 3\left(p+1\right)y^{\frac{2}{3}}+\left(3pq+p+2q-5\right)y^{\frac{1}{2}}+\left(3pq-4p-2q+2\right)y^{\frac{1}{3}}. \end{array}$$

□

By taking the first derivative of the polynomial in Theorem 14 at y=1, we obtain the first redefined Zagreb index of the chain of silicates $SC_{q}^{p}$ as follows: Let *p* be odd and p<q. Then the first redefined Zagreb index of $SC_{q}^{p}$ is $\frac{5}{2}pq+\frac{7}{6}p+\frac{1}{3}q+\frac{1}{6}$.

**Theorem** **15.** *Let p be odd and p<q. Then the second redefined Zagreb polynomial of $SC_{q}^{p}$ is $3\left(p+1\right)y^{\frac{3}{2}}+\left(3pq+p+2q-5\right)y^{2}+\left(3pq-4p-2q+2\right)y^{3}$.*

**Proof.**  Using the atom–bond partition from Table 3, in the formula of the second redefined Zagreb polynomial (7), we obtain

$$\begin{array}{ccc} ReZG_{2}\left(SC_{q}^{p},y\right) & = & \sum_{E_{3∼3}}y^{\left(\frac{3\times 3}{3+3}\right)}+\sum_{E_{3∼6}}y^{\left(\frac{3\times 6}{3+6}\right)}+\sum_{E_{6∼6}}y^{\left(\frac{6\times 6}{6+6}\right)} \end{array}$$

This gives

$$\begin{array}{ccc} ReZG_{2}\left(SC_{q}^{p},y\right) & = & 3\left(p+1\right)y^{\frac{3}{2}}+\left(3pq+p+2q-5\right)y^{2}+\left(3pq-4p-2q+2\right)y^{3}. \end{array}$$

□

By taking the first derivative of the polynomial in Theorem 15 at y=1, we obtain the second redefined Zagreb index of the chain of silicates $SC_{q}^{p}$ as follows: Let *p* be odd and p<q. Then the second redefined Zagreb index of $SC_{q}^{p}$ is $15pq-\frac{11}{2}p-2q+\frac{1}{2}$.

**Theorem** **16.** *Let p be odd and p<q. Then the third redefined Zagreb polynomial of $SC_{q}^{p}$ is $3\left(p+1\right)y^{54}+\left(3pq+p+2q-5\right)y^{196}+\left(3pq-4p-2q+2\right)y^{432}$.*

**Proof.**  Using the atom–bond partition from Table 3, in the formula of the third redefined Zagreb polynomial (8), we obtain

$$\begin{array}{ccc} ReZG_{3}\left(SC_{q}^{p},y\right) & = & \sum_{E_{3∼3}}y^{\left(3\times 3)(3+3\right)}+\sum_{E_{3∼6}}y^{\left(3\times 6)(3+6\right)}+\sum_{E_{6∼6}}y^{\left(6\times 6)(6+6\right)} \end{array}$$

This gives

$$\begin{array}{ccc} ReZG_{3}\left(SC_{q}^{p},y\right) & = & 3\left(p+1\right)y^{54}+\left(3pq+p+2q-5\right)y^{196}+\left(3pq-4p-2q+2\right)y^{432}. \end{array}$$

□

By taking the first derivative of the polynomial in Theorem 16 at y=1, we obtain the third redefined Zagreb index of the chain of silicates $SC_{q}^{p}$ as follows: Let *p* be odd and p<q. Then the third redefined Zagreb index of $SC_{q}^{p}$ is 984pq−170p+128q−554.

### Comparison

**Figure 3:**
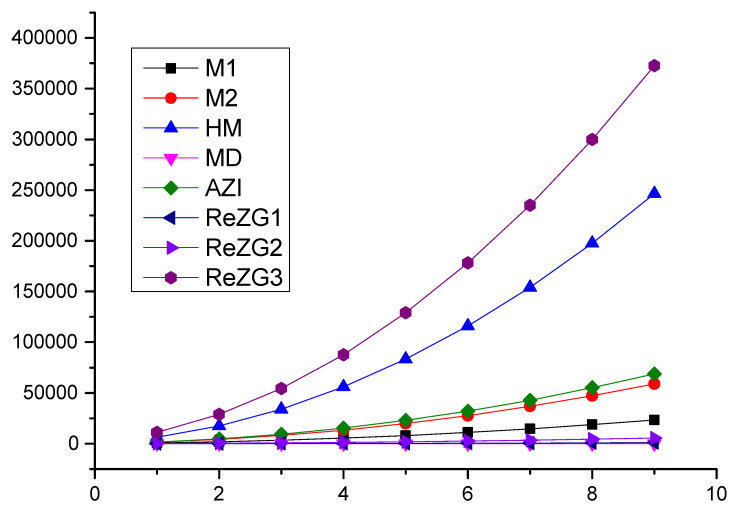
Graphical comparisons of Zagreb indices for p<q and *p* are odd.

In this section, we present a numerical comparison of the Zagreb indices and a graphical comparison of the Zagreb polynomials for p<q and *p* is odd; we use p=3,5,7,9,11,13,15,17,19 and q=4,6,8,10,12,14,16,18,20 for the chain of silicates $SC_{q}^{p}$ (Table 4, Figure 3).

## 5. Conclusions

In the analysis of quantitative structure-property relationships (QSPRs) and (QSARs), chemical indices are major implements used to approximate the characteristic features of biological activities, and physical, biomedicine, and molecular compounds. It is ordinary for questions to emerge about the characterization of silicate networks on the bases of the nature of Zagreb polynomials. We computed Zagreb polynomials for the chain of silicates under various situations in this research article. We obtained the first Zagreb, second Zagreb, hyper-Zagreb, augmented Zagreb, redefined first Zagreb, redefined second Zagreb, and redefined third Zagreb indices for the chain of silicates $SC_{q}^{p}$ from these Zagreb polynomials. For instance, topological indices or Zagreb indices are used to create quantitative structure–activity relationships (QSARs) that connect the chemical structure of molecules to the biological activities or other characteristics of such compounds.

**Open problems:** For the characterization of the chain of silicates, followers are invited to discuss or research the following open problem:

- Are Zagreb polynomials and Zagreb indices affected when both *p* and *q* are even or odd?
- The results will be interesting when p≥q.

## Figures and Tables

**Table 1:** Atom–bond partition of $SC_{q}^{p}$ for p=q.

Type of Atom–Bond	$E_{3∼3}$	$E_{3∼6}$	$E_{6∼6}$
Number of atom–bonds	3p+2	3(pq+q)−4	3(pq−2q)+2

**Table 2:** Zagreb topological indices of $SC_{q}^{p}$, for p,q≥ 2, p=q.

p	q	$M_{1}$	$M_{2}$	HM	MD	AZI	${\mathrm{ReZG}}_{1}$	${\mathrm{ReZG}}_{2}$	${\mathrm{ReZG}}_{3}$
2	2	198	396	2243	1.723	422.4811	13	46	−2744
3	3	486	1071	4278	3.3055	182.6090	27	113.5	−3646
4	4	900	2070	8392	5.388	2324.6907	46	211	−4172
5	5	1440	3393	13,856	7.9722	3848.7256	70	338.5	−4322
6	6	2106	5040	20,670	11.055	5754.7139	99	496	−4096
7	7	2898	7011	28,834	14.6388	8042.6556	133	683.5	−3494
8	8	3816	9306	38,348	18.7222	10,712.5607	172	901	−2516
9	9	4860	11,925	49,212	23.3055	13,764.3992	216	1148.5	−1162
10	10	6030	14,869	55,297	28.3888	17,198.2011	265	1426	568
11	11	7326	18,135	75,990	33.9722	21,013.9564	319	1738.5	2674
12	12	8748	21,726	89,904	40.0555	25,211.6651	378	2071	5156

**Table 3:** Atom–bond partition of $SC_{q}^{p}$; p is odd and p<q.

Type of Atom–Bond	$E_{3∼3}$	$E_{3∼6}$	$E_{6∼6}$
Number of atom bonds	3(p+1)	3pq+p+2q−5	3pq−2(2p+q−1)

**Table 4:** Zagreb indices of $SC_{q}^{p}$ for p<q and *p* is odd.

p,q	$M_{1}$	$M_{2}$	HM	MD	AZI	${\mathrm{ReZG}}_{1}$	${\mathrm{ReZG}}_{2}$	${\mathrm{ReZG}}_{3}$
3,4	661	1512	6426	4.1666	1690.6176	35	156	11,256
5,6	1746	4158	17,550	9.3333	4738.6876	83	411	28,884
7,8	3330	8100	34,074	16.5	9314.6712	151	786	54,384
9,10	5418	13,338	55,998	25.66	15,418.2684	239	1281	87,756
11,12	8010	19,872	83,322	36.83	23,049.7792	347	1896	129,000
13,14	11,106	27,702	116,044	50	32,209.1036	475	2631	178,116
15,16	14,706	36,828	154,170	65.16	42,896.2416	623	3486	235,104
17,18	18,810	47,250	197,694	82.33	55,111.1932	791	4461	299,964
19,20	23,418	58,968	246,618	101.5	68,853.9584	979	5556	372,696

## Data Availability

No data were used to support this study.

## Sample Availability

Not available.
